# Supplementary figures and images for: Serum PCSK9 is a novel serological biomarker for the diagnosis and prognosis of pancreatic cancer
Source: PeerJ. 2024 Sep 10;12:e18018. doi: 10.7717/peerj.18018 (PMC11397121; doi:10.7717/peerj.18018)

**A**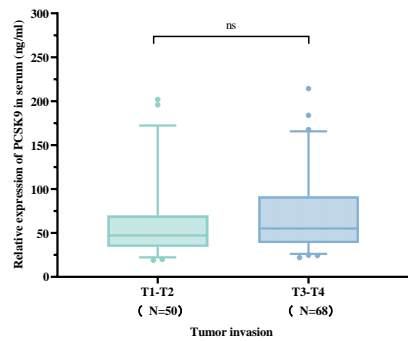**B**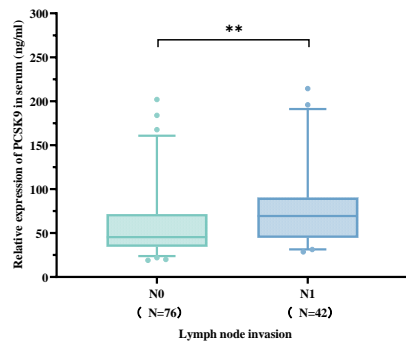**C**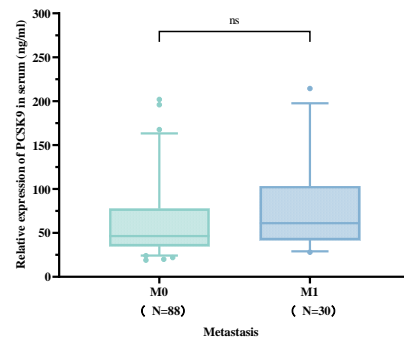**D**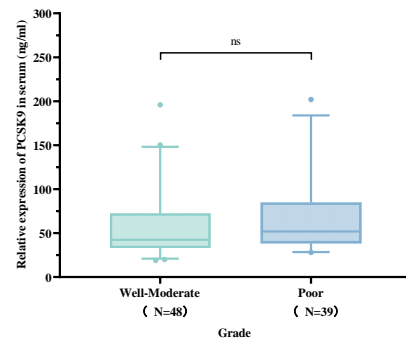**E**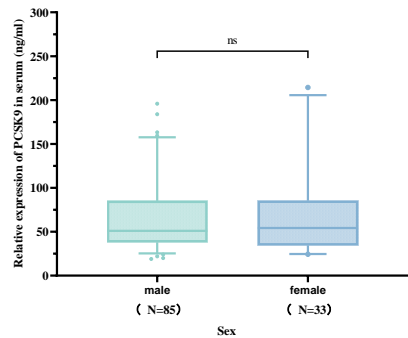**F**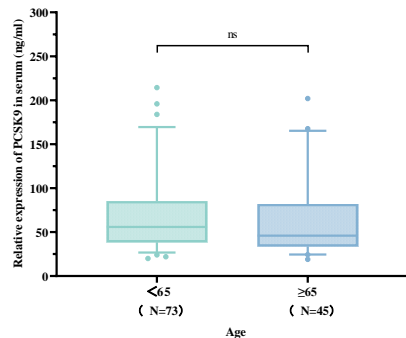**G**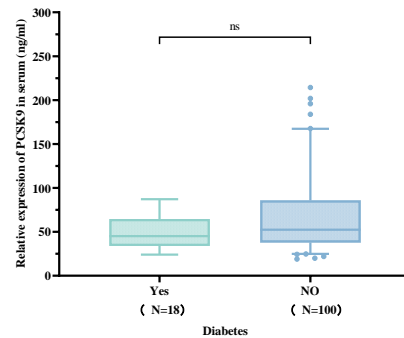**H**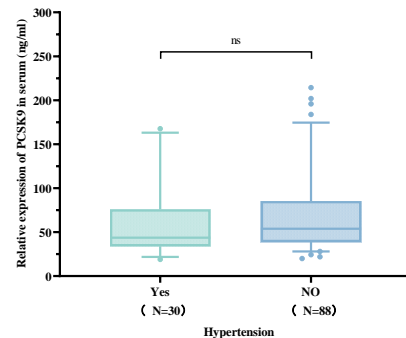

Supplement: Supplemental Information 2 — All data are presented as median (5-95 percentile). *P < 0.05, **P < 0.01 and ***P < 0.001. ns: none significance. [file peerj-12-18018-s002.pdf]
